# PredictiveNetwork: predictive gene network estimation with application to gastric cancer drug response-predictive network analysis

**DOI:** 10.1186/s12859-022-04871-z

**Published:** 2022-08-16

**Authors:** Heewon Park, Seiya Imoto, Satoru Miyano

**Affiliations:** 1 M&D Data Science Center, Tokyo Medical and Dental University, 1-5-45 Yushima, Bunkyo-ku, Tokyo, Japan; 2 Human Genome Center, Institute of Medical Science, University of Tokyo, 4-6-1 Shirokane-dai, Minato-ku, Tokyo, Japan

**Keywords:** Gene network, Anti-cancer drug sensitivity, Gastric cancer, Aldo-keto reductase family

## Abstract

**Background:**

Gene regulatory networks have garnered a large amount of attention to understand disease mechanisms caused by complex molecular network interactions. These networks have been applied to predict specific clinical characteristics, e.g., cancer, pathogenicity, and anti-cancer drug sensitivity. However, in most previous studies using network-based prediction, the gene networks were estimated first, and predicted clinical characteristics based on pre-estimated networks. Thus, the estimated networks cannot describe clinical characteristic-specific gene regulatory systems. Furthermore, existing computational methods were developed from algorithmic and mathematics viewpoints, without considering network biology.

**Results:**

To effectively predict clinical characteristics and estimate gene networks that provide critical insights into understanding the biological mechanisms involved in a clinical characteristic, we propose a novel strategy for predictive gene network estimation. The proposed strategy simultaneously performs gene network estimation and prediction of the clinical characteristic. In this strategy, the gene network is estimated with minimal network estimation and prediction errors. We incorporate network biology by assuming that neighboring genes in a network have similar biological functions, while hub genes play key roles in biological processes. Thus, the proposed method provides interpretable prediction results and enables us to uncover biologically reliable marker identification. Monte Carlo simulations shows the effectiveness of our method for feature selection in gene estimation and prediction with excellent prediction accuracy. We applied the proposed strategy to construct gastric cancer drug-responsive networks.

**Conclusion:**

We identified gastric drug response predictive markers and drug sensitivity/resistance-specific markers, *AKR1B10*, *AKR1C3*, *ANXA10*, and *ZNF165*, based on GDSC data analysis. Our results for identifying drug sensitive and resistant specific molecular interplay are strongly supported by previous studies. We expect that the proposed strategy will be a useful tool for uncovering crucial molecular interactions involved a specific biological mechanism, such as cancer progression or acquired drug resistance.

**Supplementary Information:**

The online version contains supplementary material available at 10.1186/s12859-022-04871-z.

## Background

Gene networks are crucial for understanding complex disease mechanisms because the molecular mechanisms involved in disease are related to abnormalities in complex molecular networks, rather than in a single gene. To estimate gene regulatory networks, various computational strategies have been proposed and used to identify the molecular interplay involved in specific biological processes (e.g., cancer progression or anti-cancer drug sensitivity). Estimated gene networks have been applied to uncover complex disease mechanisms and identify drug-response marker. Importantly, the effectiveness of network-based analysis has been validated [1, 2]. The gene regulatory network has been also applied to predict a specific clinical characteristic, e.g., cancer prediction, pathogenicity prediction, where the network is used as an input of prediction model [3–5]. In recent years, predicting drug sensitivity and understanding the molecular mechanisms related to drug resistance in cancer cells has drawn a large amount of attention. Several studies predicted drug sensitivity based on gene networks, e.g., protein-protein interaction (PPI) networks or prior knowledge networks [6–8].

Although several computational strategies have been developed for network-based prediction, existing studies estimate gene networks first, and predict a specific clinical characteristic based on pre-estimated networks. Thus, we cannot identify the gene regulatory system characteristics that are related to a specific clinical characteristic (i.e., the object of prediction). Furthermore, existing studies focus only on algorithmic and statistical performance and develop prediction strategies purely from mathematical and computational viewpoints without considering network biology. This leads to difficulty when interpreting the prediction results and when identifying biomarkers.

To address this issue, we propose a novel statistical strategy called a PredictiveNetwork. We consider the response variable-predictive gene network estimation, where the response variable is a specific clinical characteristic. PredictiveNetwork performs loss-of-function analysis for gene network estimation and prediction. The objective function of PredictiveNetwork consists of loss functions for gene network estimation and prediction, and thus the gene network estimation and prediction are simultaneously performed. In our strategy, the gene network is estimated by minimizing the losses of estimating gene regulatory systems and response variable prediction, and thus we can uncover prediction specific gene regulatory system, i.e., a prediction-specific gene regulatory network for a clinical characteristic. Furthermore, we incorporate knowledge of network biology into the statistical prediction model based on the network constraint $L_{1}$-type regularization, as described in [9, 10]. Genes linked within networks/pathways may have similar biological functions, while hub genes, which interact with many other genes, are crucial markers that play key roles in gene regulation and biological processes [11]. In our strategy, differences in coefficients for neighboring genes in the network are smoothed, followed by simultaneous selection of the related genes. We also encourage that hub genes would have large coefficients and/or would be easily selected in the prediction model. In short, the prediction model is constructed based on crucial subnetworks consisting of hub genes and their target/regulator genes. Thus, we can perform biologically reliable interpretation of the prediction results based on molecular interplay in the subnetwork and reliably identify biomarkers (e.g., uncovering crucial genes and molecular interactions involved in a specific biological process).

We demonstrate the effectiveness of the PredictiveNetwork for prediction and feature selection accuracies in the prediction model and edge selection accuracies in gene network estimation using Monte Carlo simulations. We also applied our strategy to the Sanger Genomics of Drug Sensitivity in Cancer (GDSC) dataset from the Cancer Genome Project to construct drug response-predictive gene network. We performed drug sensitivity prediction and sensitivity-predictive gene network analysis for the FDA-approved gastric cancer drugs, doxorubicin, mitomycin-c, 5-Fluorouracil (5-FU), and docetaxel. Our strategy shows effective prediction results for mitomycin-c, 5-Fluorouracil, and docetaxel sensitivity. Then, we identified predictive drug response markers and characteristics of the associated regulatory systems in drug-sensitive and -resistant cell lines. More than half of the identified gastric cancer drug response markers have strong evidences as biomarkers for gastric cancer and anti-cancer drug responses. In particular, the identified *AKR* family (*AKR1C1*, *AKR1C3*, *AKR1B10*) are likely anti-cancer drug resistance markers. We identified *AKR1C3* and *AKR1B10* as having drug resistance characteristics and that their hubness becomes significantly smaller in drug-resistant compared to drug-sensitive cell lines. For the drug sensitive cell lines, activities of *ANXA10* and *ZNF165* are identified as characteristics and the hubness of *ANXA10* in drug-sensitive cell lines is strongly supported by previous studies. Further, *ZNF165* may be a novel marker of gastric cancer drug responsiveness. Our results of GDSC data analysis suggest that the molecular interplay between genes of the AKR family, rather than single genes alone, plays key roles in acquired gastric cancer drug resistance. Thus, suppressors of AKR family genes and inducers of *ANXA10/ZNF165* may reduce drug resistance of cancer cell lines.

The remainder of this paper is organized as follows: In the “Method” section, we propose a novel strategy for predictive gene network estimation and then describe the numerical solution of the PredictiveNetwork. Then, we show the results of simulation studies in the “Monte Carlo simulations” section. Finally, we describe the results of gastric cancer drug response-predictive gene network analysis. Conclusions are provided in the “Discussion” section.

## Method

Suppose ${\varvec{X}}=({\varvec{x}}_{1},...,{\varvec{x}}_{n})^{T} \in {\mathbb {R}}^{n\times p}$ is an $n \times p$ data matrix describing the expression of *p* possible regulators that control target gene transcription ${\varvec{y}}_{j} \in {\mathbb {R}}^{n}, j=1,...,k$. Consider the linear regression model,

1 $$\begin{aligned} {\varvec{y}}_{j}=\sum _{l=1}^{p}\beta _{jl}{\varvec{x}}_{l}+\varvec{\epsilon }_{j}, \quad j=1,...,k, \end{aligned}$$

where $\beta _{jl}$ is the regression coefficient that represents the effect of each regulator ${\varvec{x}}_{j}$ on its target ${\varvec{y}}_{j}$ and $\varvec{\epsilon }_{j}=(\epsilon _{j1},...,\epsilon _{jn})^{T}$ is a random error vector that is assumed to be independently and identically distributed with mean 0 and variance $\sigma ^{2}_{y}$.

Gene regulatory networks are often estimated using the following $L_{1}$-type regularization methods (e.g., lasso, elastic net, fused lasso, etc.) [12–14],

2 $$\begin{aligned} \varvec{\beta }_{j}=\mathop {\mathrm{arg~min}}\limits _{\varvec{\beta }_{j}}\left\{ \sum _{i=1}^{n}(y_{ij}-\sum _{l=1}^{p}x_{il} \beta _{jl})^{2}+P_{\delta ,\lambda }(\varvec{\beta }_{j})\right\} , \end{aligned}$$

where

$$\begin{aligned} P(\varvec{\beta }_{j})=\lambda _{1}\sum _{l=1}^{p}\frac{1}{2}\beta ^2_{jl}+\lambda _{2}\sum _{l=1}^{p}|\beta _{jl}|, \end{aligned}$$

and $\lambda _1, \lambda _2>0$ are the regularization parameters of $\varvec{\beta }$.

Although several statistical methods were developed for network-based prediction models, previous studies performed network estimation and prediction separately, i.e. the gene network was constructed first and then the estimated network was used as the prediction model input [6–8]. Furthermore, existing prediction models were developed purely from an algorithmic and statistical point of view, without considering network biology. Thus, the existing methods cannot provide effective interpretation of the prediction results, which causes difficulty in reliable biomarker identification.

### Predictive gene regulatory network estimation

To effectively predict a specific biological mechanism and gene networks estimation that provides critical insights into understanding biological mechanisms, we propose a novel statistical method, called a PredictiveNetwork.

Suppose we have *n* independent observations for the response variables ${\varvec{z}}=({z_{1},...,z_{n}})^{T}$. We consider response variable-predictive gene network estimation and propose a novel model that enables us to simultaneously estimate gene network and predict the response variable,

3 $$\begin{aligned}&\mathop {\mathrm{arg~min}}\limits _{{\varvec{B}},{ \varvec{\theta }}}\left\{ \sum _{i=1}^{n}(z_{i}-\theta _{0}-\varvec{\theta }^{T}{\varvec{B}}^{T}{\varvec{x}}_{i})^{2} +\sum _{j=1}^{k}\sum _{i=1}^{n}||y_{ij}-\varvec{\beta }^{T}_{j}{\varvec{x}}_{i}||^{2}\right. \nonumber \\&\quad \left. +\lambda _{1}\sum _{j=1}^{k}||\varvec{\beta }_{j}||+\lambda _{2}\sum _{j=1}^{k}||\varvec{\beta }_{j}||^2+\lambda _{3}\sum _{j=1}^{k}||\varvec{\theta }_{j}|| \right\} \end{aligned}$$

where $\theta _{0}$ is an intercept, $\varvec{\theta }=(\theta _{1},...,\theta _{k})^{T}$ is a coefficient vector of gene expression levels for the response variable ${\varvec{z}}$, ${\varvec{B}}=(\varvec{\beta }_{1},...,\varvec{\beta }_{k})$ is a $p \times k$ matrix describing regulatory systems between genes in the directional network, and $\lambda _{3}>0$ is a regularization parameter to impose sparsity on $\varvec{\theta }$ in the prediction model. The first term in (3) indicates the loss function for the prediction of a response variable based on the gene network and the second term is the loss function of gene network estimation.

In the proposed method, the regularity system between genes ${\varvec{B}}$ is estimated to minimize errors for both network estimation and prediction. In other words, our method can provide prediction-specific network estimation results. Thus, we can identify crucial information for understanding biological mechanisms (i.e., response variables in the prediction model) based on the estimated network.

To achieve more biologically interpretable prediction results, we incorporate network biology into the prediction model using network constraint regularization [9, 10]. The estimated network from the second term in (3) can be represented by a weighted graph $G=(V,E,W)$, where $V=\{1,...,p\}$ is the set of vertices corresponding to *p* genes and $E \in V \times V$ is the set of edges. (*i*, *j*) indicates a link between vertices *i* and *j* (i.e., genes *i* and *j*) and $W=(w_{ij}), (i,j) \in E$ is the edge weight. The normalized Laplacian matrix ${\varvec{L}}$ for the graph is given as *G* [9, 10],

4 $$\begin{aligned} {\varvec{L}}=l_{ij}= {\left\{ \begin{array}{ll} 1-\frac{w_{ij}}{d_{i}} &{} \text {if } i=j \text { and } d{_i}\ne 0,\\ -\frac{w_{ij}}{\sqrt{d_{i}d_{j}}} &{} \text {if } (i,j)\in E,\\ 0 &{} \text {otherwise} \end{array}\right. } \end{aligned}$$

where $d_{i}$ is the degree of each gene, which is given as $d_{i}=\sum _{i\sim j}^{w_{ij}}$.

In our method, the effect of regulators on their target genes are estimated in ${\varvec{B}}$, where the rows and columns of ${\varvec{B}}$ indicate the regulator and target gene, respectively. Thus, we compute ${\varvec{W}}=w_{ij}$ based of effect of the *i*th gene to *j*th gene (i.e., $B_{ij}$) and the *j*th gene to *i*th gene (i.e., $B_{ji}$)as follows,

5 $$\begin{aligned} {\varvec{W}}=w_{ij}=\frac{|B_{ij}|+|B_{ij}|}{2} \end{aligned}$$

We describe the estimated gene network based on the Laplacian matrix and incorporate the estimated network into the prediction model based on ${\varvec{L}}$. We then propose PredictiveNetwork as follows,

6 $$\begin{aligned}&\mathop {\mathrm{arg~min}}\limits _{{\varvec{B}},\varvec{\theta }}\left\{ \sum _{i=1}^{n}(z_{i}-\theta _{0} -\varvec{\theta }^{T}{\varvec{B}}^{T}{\varvec{x}}_{i})^{2} +\sum _{j=1}^{k}\sum _{i=1}^{n}||y_{ij}-\varvec{\beta }^{T}_{j}{\varvec{x}}_{i}||^{2} \right\} \nonumber \\&\quad +\lambda _{1}\sum _{j=1}^{k}||\varvec{\beta }_{j}||+\lambda _{2}\sum _{j=1}^{k}||\varvec{\beta }_{j}||^2 +\lambda _{3}||\varvec{\theta }||+\lambda _{4}\varvec{\theta }^{T}{\varvec{L}}^{a}\varvec{\theta }\nonumber \\&=\mathop {\mathrm{arg~min}}\limits _{{\varvec{B}},\varvec{\theta }}\left\{ \sum _{i=1}^{n}(z_{i}-\theta _{0}-\varvec{\theta }^{T}{\varvec{B}}^{T}{\varvec{x}}_{i})^{2} +\sum _{j=1}^{k}\sum _{i=1}^{n}||y_{ij}-\varvec{\beta }^{T}_{j}{\varvec{x}}_{i}||^{2}\right. \nonumber \\&\quad \left. +\lambda _{1}\sum _{j=1}^{k}||\varvec{\beta }_{j}||+\lambda _{2}\sum _{j=1}^{k}||\varvec{\beta }_{j}||^2 +\lambda _{3}||\varvec{\theta }||+\lambda _{4}\sum _{q=1}^{k}\sum _{j=1}^{k}\left( \frac{\text {sgn}(\theta _{q})\theta _{q}}{\sqrt{d_{q}}}-\frac{\text {sgn}(\theta _{j})\theta _{j}}{\sqrt{d_{j}}}\right) ^{2}w_{qj}\right\} , \end{aligned}$$

where ${\varvec{L}}^{a}={\varvec{S}}^{T}{\varvec{L}}{\varvec{S}}$ with ${\varvec{S}}=\text {diag}(\text {sgn}({\hat{\theta }}_{1}),...,\text {sgn}({\hat{\theta }}_{k}))$. The last term in (6) penalizes the differences of the scaled coefficients between the neighboring genes in the network. From the following local quadratic approximation of $L_{1}$-type penalty [15],

7 $$\begin{aligned} |\theta _{j}|\approx |{\tilde{\theta }}_{j}|+\text {sgn}({\tilde{\theta }}_{j})(\theta _{j} -{\tilde{\theta }}_{j})=\text {sgn}({\tilde{\theta }}_{j})\theta _{j} \quad \text {for}\quad \theta _{j}\approx {\tilde{\theta }}_{j}, \end{aligned}$$

the last term in (6) can be represented as [10]

8 $$\begin{aligned}&\lambda _{4}\sum _{q=1}^{k}\sum _{j=1}^{k}{(}\frac{\text {sgn}(\theta _{q})\theta _{q}}{\sqrt{d_{q}}} -\frac{\text {sgn}(\theta _{j})\theta _{j}}{\sqrt{d_{j}}}{)}^{2}w_{qj}\nonumber \\&\quad =\lambda _{4}\sum _{q=1}^{k}\sum _{j=1}^{k}{(}\frac{|\theta _{q}|}{\sqrt{d_{q}}} -\frac{|\theta _{j}|}{\sqrt{d_{j}}}{)}^{2}w_{qj}. \end{aligned}$$

The genes linked in the networks may have similar biological functions. Thus, we encourage similarity in gene coefficients in the prediction model by using the network constrained penalty. The penalty term enables us to locally smooth the network and encourage the simultaneous selection of related variables, even though neighboring genes have opposite coefficient signs. The hub genes that have many interactions with other genes play a key role in gene regulation and biological processes [11], and thus are crucial markers to understand specific biological mechanisms. In our model, a relatively small penalty is imposed on the hub genes of the estimated networks by re-scaling the coefficients with the square root of the degrees. This scaling causes the hub genes and their regulator/target genes to have relatively large coefficients and/or be easily selected by penalties on the coefficients between neighboring genes. Thus, our method constructs the prediction model based on crucial sub networks, which leads to effective interpretation of the prediction results and predictive marker identification using network biology.

In our model, the gene network described by ${\varvec{B}}$ is estimated to minimize error for not only network estimation but also prediction. It implies that the network is estimated to be optimized for explain the response variable ${\varvec{z}}$. Thus, we can effectively uncover the biological mechanism for the response variable based on the estimated gene regulatory network. Furthermore, the coefficient $\varvec{\theta }$ explains the change in a response variable as changes of the effect of $l^{th}$ regulator gene on $j^{th}$ target gene (i.e., ${\varvec{x}}_{l}{\hat{\beta }}_{jl}$). That is, the change in specific-biological characteristics (e.g., drug sensitivity of cell lines) is explained by the regulatory system between genes. In short, we can interpret the complex mechanism of disease based on not a single gene but molecular interplays between genes, and it leads to biologically reliable interpretation.

### Implementation

To simultaneously perform prediction and gene network estimation, we adapted the following coordinate descent algorithm for estimating ${\varvec{B}}$ and $\varvec{\theta }$ [16, 17].

*Step 1.*: The optimization of $\beta _{jl}$ in (6), given $\varvec{\theta }$ and $\theta _{0}$ has the following solution,
  9 $$\begin{aligned} {\hat{\beta }}_{jl}\leftarrow \frac{S(\sum _{i=1}^{n}x_{il}\{\theta _{j}(z_{i}-z_{i}^{(jl)})+(y_{ij}-y^{(l)}_{ij})\}, \frac{1}{2}\lambda _{1})}{\sum _{i=1}^{n}x^{2}_{il}(\theta ^{2}_{j}+1)+\lambda _{2}}\quad l=1,...,p;\quad j=1,...,k, \end{aligned}$$
  where
  $$\begin{aligned}&z^{(jl)}_{i}=\theta _{0}+\sum _{j=1}^{k}\sum _{r\ne l}\theta _{j}\beta _{jr}x_{ir}+\sum _{g\ne j}\theta _{g}\beta _{gl}x_{il},\quad j=1,...,k\\&y^{(l)}_{ij}=\sum _{r \ne l}\beta _{jr}x_{ir}, \end{aligned}$$
  and $S(\theta ,\lambda )$ is a soft thresholding operator with value
  10 $$\begin{aligned} S(\theta ,\lambda )= {\left\{ \begin{array}{ll} \theta -\lambda &{} \text {if } \theta >0 \text { and } \lambda<|\theta |,\\ \theta +\lambda &{} \text {if } \theta<0 \text { and } \lambda <|\theta |,\\ 0 &{} \text {if } \lambda \ge |\theta |.\\ \end{array}\right. } \end{aligned}$$
*Step 2.*: We then compute ${\varvec{W}}$ and ${\varvec{L}}$ based on (4) and (5). The coordinate-wise update of $\theta _{j}$ given ${\varvec{B}}$, ${\varvec{L}}$, and $\theta _{0}$ has the following form,
  11 $$\begin{aligned} {\hat{\theta }}_{j}&\leftarrow \frac{S(\sum _{i=1}^{n}\varvec{\beta }^{T}_{j}{\varvec{x}}_{i}(z_{i}-z^{(j)}_{i})-\lambda _{4}\sum _{q \ne j}l^{a}_{qj}\theta _{q},\frac{\lambda _{3}}{2})}{\sum _{i=1}^{n}(\varvec{\beta }^{T}_{j}{\varvec{x}}_{i})^{2}+\lambda _{4}L^{a}_{jj}}, \end{aligned}$$
  where $$z^{(j)}_{i}=\theta _{0}+\sum _{g\ne j}\theta _{g}\varvec{\beta }^{T}_{g}{\varvec{x}}_{i}$$.
*Step 3.*: The estimate of $\theta _{0}$ given ${\varvec{B}}$ and $\varvec{\theta }$ is given as
  12 $$\begin{aligned} {\hat{\theta }}_{0}=\frac{1}{n}\sum _{i=1}^{n}\left( z_{i}-\sum _{j=1}^{k}\theta _{j}\sum _{l=1}^{p}\beta _{jl}x_{il}\right) . \end{aligned}$$
*Step 4.*: Finally, we update the parameters $\hat{{\varvec{B}}}, \hat{\varvec{\theta }}, \hat{\theta _{0}}$ cyclically until convergence.

### Covariance updates for computational efficiency

The proposed method updates $\varvec{\theta }$ for *p* variables and $\beta _{jl}$ for $p \times k$ variables, simultaneously. This implies that the PredictiveNetwork suffers from computational complexity. Since gene expression data usually consists of a large number of features, the predictive gene network estimation requires a huge computational complexity. To reduce this computational complexity, we considered covariance updates [16].

The coordinate update of ${\varvec{B}}$ in (9) can be rewritten as follows:

13 $$\begin{aligned}&\sum _{i=1}^{n}x_{il}\left\{ \theta _{j}\left( z_{i}-z^{(jl)}_{i}\right) +\left( y_{ij}-y^{(l)}_{ij}\right) \right\} \nonumber \\&=\sum _{i=1}^{n}x_{il}\left\{ \theta _{j}\left( r^{z}_{i}+\theta _{j}{\tilde{\beta }}_{jl}x_{il}\right) +\left( r^{y}_{ij}+{\tilde{\beta }}_{jl}x_{il}\right) \right\} \nonumber \\&={\tilde{\beta }}_{jl}\sum _{i=1}^{n}x^{2}_{il}\left( \theta ^{2}_{j}+1\right) +\theta _{j}\sum _{i=1}^{n}x_{il}r^{z}_{i}+\sum _{i=1}^{n}x_{il}r^{y}_{ij} \end{aligned}$$

where ${\tilde{\beta }}_{jl}$ is the estimate of $\beta _{jl}$ obtained from the previous update, $r^{z}_{i}=z_{i}-\theta _{0}-\sum _{j=1}^{k}\sum _{r=1}^{p}\theta _{j}{\tilde{\beta }}_{jr}x_{ir}$, and $r^{y}_{ij}=y_{ij}-\sum _{r=1}^{p}{\tilde{\beta }}_{jr}x_{il}$. The second term of (13) can be represented

14 $$\begin{aligned} \theta _{j}\sum _{i=1}^{n}x_{il}r^{z}_{i}&=\theta _{j}\left( \sum _{i=1}^{n}x_{il}z_{i}-\theta _{0} \sum _{i=1}^{n}x_{il}-\sum _{j=1}^{k}\sum _{r=1}^{p}\theta _{j}{\tilde{\beta }}_{jr}{\varvec{x}}^{T}_{r}{\varvec{x}}_{l}\right) \nonumber \\&=\theta _{j}\left( \sum _{i=1}^{n}x_{il}z_{i}-\theta _{0}\sum _{i=1}^{n}x_{il} -\sum _{j,r:|{\tilde{\beta }}_{jr}|>0}\theta _{j}{\tilde{\beta }}_{jr}{\varvec{x}}^{T}_{r}{\varvec{x}}_{l}\right) \end{aligned}$$

and the third term is given as

15 $$\begin{aligned} \sum _{i=1}^{n}x_{il}r^{y}_{ij}&=\sum _{i=1}^{n}x_{il}y_{ij} -\sum _{r=1}^{p}{\tilde{\beta }}_{jr}{\varvec{x}}^{T}_{r}{\varvec{x}}_{l}\nonumber \\&=\sum _{i=1}^{n}x_{il}y_{ij}-\sum _{r:|{\tilde{\beta }}_{jr}|>0}{\tilde{\beta }}_{jl}{\varvec{x}}^{T}_{r}{\varvec{x}}_{l}. \end{aligned}$$

This implies that only the last terms of (14) and (15) are updated for ${\tilde{\beta }}_{jl}\ne 0$. Thus, we can reduce computational complexity for estimating ${\varvec{B}}$.

To estimate $\varvec{\theta }$, part of the update in (11) can be rewritten as

16 $$\begin{aligned}&\sum _{i=1}^{n}\varvec{\beta }^{T}_{j}{\varvec{x}}_{i}(z_{i}-z^{(j)}_{i})\nonumber \\&=\sum _{i=1}^{n}\varvec{\beta }^{T}_{j}{\varvec{x}}_{i}z_{i} -\theta _{0}\sum _{i=1}^{n}\varvec{\beta }^{T}_{j}{\varvec{x}}_{i} -\sum _{i=1}^{n}\varvec{\beta }^{T}_{j}{\varvec{x}}_{i}\sum _{r=1}^{k} {\tilde{\theta }}_{j}\varvec{\beta }^{T}_{j}{\varvec{x}}_{i} +{\tilde{\theta }}_{j}\sum _{i=1}^{n}(\varvec{\beta }^{T}_{j}{\varvec{x}}_{i})^{2}\nonumber \\&=\sum _{i=1}^{n}\varvec{\beta }^{T}_{j}{\varvec{x}}_{i}z_{i}-\theta _{0} \sum _{i=1}^{n}\varvec{\beta }^{T}_{j}{\varvec{x}}_{i} -\sum _{r:|{\tilde{\theta }}_{r}|>0}{\tilde{\theta }}_{j}({\varvec{X}} \varvec{\beta }_{j})^{T}({\varvec{X}}\varvec{\beta }_{r}) +{\tilde{\theta }}_{j}\sum _{i=1}^{n}(\varvec{\beta }^{T}_{j}{\varvec{x}}_{i})^{2}, \end{aligned}$$

where ${\tilde{\theta }}_{j}$ is the estimate of $\theta _{j}$ obtained from previous update. We update the third term of (16) only when ${\tilde{\theta }}_{j} \ne 0$, which reduces the computational complexity for estimating $\varvec{\theta }$.

## Monte Carlo simulations

Monte Carlo simulations were conducted to investigate the performance of the proposed method. We consider simulation scenarios by benchmark of previous studies on the network-based regularization [9, 10]. We simulated gene expression data under the assumed network. We supposed that each transcription factor gene (TF) regulates 10 genes and the TF expression levels are generated from a standard normal distribution.

The expression levels of each of the regulated genes (${\varvec{y}}_{j}$, $j=1,...10$) of the TF (${\varvec{x}}_{t}$) were generated based on the expression level of $t^{th}$ TF as follows,

$$\begin{aligned} {\varvec{y}}_{j}=\beta _{jt}{\varvec{x}}_{t}+\epsilon ^{y}_{j}, \quad j=1,...,10. \end{aligned}$$

where $$\epsilon ^{y}_{j}\sim N(0,\sigma _{y}^2)$$.

The response variable ${\varvec{z}}$ is generated based on the regulatory effect of genes, i.e., based on the gene expression levels ${\varvec{X}}$ and the effect of regulators on targets ${\varvec{B}}=(\varvec{\beta }_{1},...,\varvec{\beta }_{k})$ as follows,

$$\begin{aligned} {\varvec{z}}={\varvec{X}}{\varvec{B}}\varvec{\theta }+\varvec{\epsilon }^{z}, \end{aligned}$$

where $$\varvec{\epsilon }^{z}\sim N(0,\sigma ^{2}_{z})$$.

For response variable predictive gene network estimation, we considered the following four scenarios.

Scenario 1: $$\begin{aligned}&\beta _{jt}=0.95, \quad j=1,...,10,\quad t=1,...,T\\&\varvec{\theta }=(1,\underbrace{\frac{1}{\sqrt{10}},..., \frac{1}{\sqrt{10}}}_{10},-1,\underbrace{\frac{-1}{\sqrt{10}},..., \frac{-1}{\sqrt{10}}}_{10},0.8,\underbrace{\frac{0.8}{\sqrt{10}},..., \frac{0.8}{\sqrt{10}}}_{10},-0.8,\underbrace{\frac{-0.8}{\sqrt{10}},..., \frac{-0.8}{\sqrt{10}}}_{10},0,...,0) \end{aligned}$$
Scenario 2: $$\begin{aligned}&\beta _{jt}=0.95,\quad j=1,...,5,\quad t=1,...,T\\&\beta _{jt}=0.80,\quad j=6,...,10,\quad t=1,...,T\\&\varvec{\theta }=(1,\underbrace{\frac{1}{\sqrt{10}},..., \frac{1}{\sqrt{10}}}_{10},-1,\underbrace{\frac{-1}{\sqrt{10}},..., \frac{-1}{\sqrt{10}}}_{10},0.8,\underbrace{\frac{0.8}{\sqrt{10}},..., \frac{0.8}{\sqrt{10}}}_{10},-0.8,\underbrace{\frac{-0.8}{\sqrt{10}},..., \frac{-0.8}{\sqrt{10}}}_{10},0,...,0) \end{aligned}$$
Scenario 3: $$\begin{aligned}&\beta _{jt}=0.95, \quad j=1,...,10,\quad t=1,...,T\\&\varvec{\theta }=(1,\underbrace{\frac{1}{\sqrt{5}},..., \frac{1}{\sqrt{5}}}_{10},-1,\underbrace{\frac{-1}{\sqrt{15}},..., \frac{-1}{\sqrt{15}}}_{10},0.8,\underbrace{\frac{0.8}{\sqrt{5}},..., \frac{0.8}{\sqrt{5}}}_{10},-0.8,\underbrace{\frac{-0.8}{\sqrt{15}},..., \frac{-0.8}{\sqrt{15}}}_{10},0,...,0) \end{aligned}$$
Scenario 4: $$\begin{aligned}&\beta _{jt}=0.95,\quad j=1,...,5,\quad t=1,...,T\\&\beta _{jt}=0.80,\quad j=6,...,10,\quad t=1,...,T\\&\varvec{\theta }=(1,\underbrace{\frac{1}{\sqrt{5}},..., \frac{1}{\sqrt{5}}}_{10},-1,\underbrace{\frac{-1}{\sqrt{15}},..., \frac{-1}{\sqrt{15}}}_{10},0.8,\underbrace{\frac{0.8}{\sqrt{5}},..., \frac{0.8}{\sqrt{5}}}_{10},-0.8,\underbrace{\frac{-0.8}{\sqrt{15}},..., \frac{-0.8}{\sqrt{15}}}_{10},0,...,0) \end{aligned}$$

The scenarios 1 and 2 are adapted from the works of Li and Li [9]. In order to consider different edge size of regulator on their target genes, which is reasonable for network biology, we also perform simulation studies based on scenarios 3 and 4 in line with Sun et al. [10].

We considered $\sigma _{y}^2=0.5$ and simulated 50 datasets consisting of $n=200$ observations from the 4 scenarios, where the training, validation, and test datasets consisted of 80% (160), 10% (20) and 10% (20) observations, respectively.

**Table 1:** Results: $$\sigma _{z}=0.1$$

No.TFs	Scenario	Feature selection of genes											
		TP				TN				Ave			
		Pro	NW.P	EL	LA	Pro	NW.P	EL	LA	Pro	NW.P	EL	LA
5	1	0.99	0.65	0.41	0.39	0.97	0.82	0.86	0.86	**0**.**98**	0.74	0.64	0.63
	2	0.98	0.69	0.40	0.39	0.96	0.80	0.87	0.89	**0**.**97**	0.74	0.63	0.64
	3	0.99	0.69	0.39	0.39	0.94	0.84	0.91	0.89	**0**.**96**	0.77	0.65	0.64
	4	0.98	0.65	0.42	0.40	0.95	0.87	0.82	0.85	**0**.**97**	0.76	0.62	0.62
10	1	0.99	0.66	0.37	0.37	0.96	0.90	0.93	0.91	**0**.**97**	0.78	0.65	0.64
	2	0.98	0.69	0.35	0.34	0.95	0.87	0.92	0.93	**0**.**97**	0.78	0.63	0.63
	3	0.99	0.66	0.36	0.35	0.96	0.92	0.92	0.93	**0**.**97**	0.79	0.64	0.64
	4	0.98	0.68	0.35	0.33	0.96	0.87	0.92	0.92	**0**.**97**	0.78	0.63	0.63
25	1	0.99	0.69	0.30	0.29	0.96	0.99	0.98	0.99	**0**.**98**	0.84	0.64	0.64
	2	0.98	0.72	0.28	0.28	0.96	0.99	0.98	0.98	**0**.**97**	0.85	0.63	0.63
	3	0.99	0.71	0.28	0.28	0.96	0.99	0.99	0.99	**0**.**98**	0.85	0.63	0.63
	4	0.99	0.69	0.30	0.30	0.96	0.99	0.98	0.98	**0**.**97**	0.84	0.64	0.64
50	1	0.99	0.75	0.32	0.31	0.96	0.99	0.98	0.98	**0**.**98**	0.87	0.65	0.64
	2	0.98	0.71	0.31	0.30	0.97	0.99	0.98	0.98	**0**.**97**	0.85	0.64	0.64
	3	0.99	0.75	0.28	0.28	0.97	0.99	0.99	0.99	**0**.**98**	0.87	0.63	0.63
	4	0.99	0.74	0.28	0.27	0.97	0.99	0.98	0.98	**0**.**98**	0.87	0.63	0.63
100	1	0.99	0.78	0.29	0.29	0.97	0.99	0.99	0.99	**0**.**98**	0.88	0.64	0.64
	2	0.98	0.75	0.27	0.28	0.97	0.99	0.99	0.98	**0**.**97**	0.87	0.63	0.63
	3	0.99	0.83	0.28	0.27	0.97	0.99	0.99	0.99	**0**.**98**	0.91	0.63	0.63
	4	0.98	0.73	0.27	0.27	0.97	0.99	0.99	0.99	**0**.**98**	0.86	0.63	0.63

No.TFs	Scenario	Feature selection of edges						Prediction accuracy					
		TP		TN		Ave		MSE					
		Pro	NW.P	Pro	NW.P	Pro	NW.P	Pro	NW.P	EL	LA	XGB	NN
5	1	1.00	1.00	1.00	0.92	1.00	0.96	0.117	0.127	0.112	**0**.**110**	9.262	2.460
	2	1.00	1.00	1.00	0.92	1.00	0.96	0.111	0.117	**0**.**107**	0.108	8.495	2.518
	3	1.00	1.00	1.00	0.92	1.00	0.96	**0**.**111**	0.125	0.120	0.119	11.243	3.141
	4	1.00	1.00	1.00	0.92	1.00	0.96	**0**.**106**	0.118	0.121	0.120	9.991	3.105
10	1	1.00	1.00	1.00	0.96	1.00	0.98	**0**.**117**	0.132	0.123	0.122	9.728	3.466
	2	1.00	1.00	1.00	0.96	1.00	0.98	0.119	0.127	**0**.**104**	**0**.**104**	8.307	3.597
	3	1.00	1.00	1.00	0.96	1.00	0.98	**0**.**111**	0.116	0.121	0.120	11.034	4.159
	4	1.00	1.00	1.00	0.96	1.00	0.98	**0**.**108**	0.117	0.114	0.114	9.475	4.416
25	1	1.00	1.00	1.00	0.98	1.00	0.99	**0**.**117**	0.134	0.126	0.126	10.753	5.686
	2	1.00	1.00	1.00	0.98	1.00	0.99	**0**.**113**	0.129	0.121	0.120	9.192	5.666
	3	1.00	1.00	1.00	0.98	1.00	0.99	0.121	0.144	**0.117**	**0**.**117**	11.614	7.257
	4	1.00	1.00	1.00	0.98	1.00	0.99	**0**.**124**	0.146	0.134	0.133	10.439	7.161
50	1	1.00	1.00	1.00	0.99	1.00	1.00	**0**.**120**	0.140	0.128	0.128	10.641	11.463
	2	1.00	1.00	1.00	0.99	1.00	1.00	**0.117**	0.133	0.122	0.122	9.678	11.250
	3	1.00	1.00	1.00	0.99	1.00	1.00	0.131	0.152	0.125	**0**.**124**	11.618	14.328
	4	1.00	1.00	1.00	0.99	1.00	1.00	**0**.**111**	0.132	0.120	0.120	10.376	14.048
100	1	1.00	1.00	1.00	1.00	1.00	1.00	0.122	0.133	**0**.**114**	**0**.**114**	12.358	23.335
	2	1.00	1.00	1.00	1.00	1.00	1.00	**0**.**128**	0.134	0.130	0.129	11.206	21.715
	3	1.00	1.00	1.00	1.00	1.00	1.00	**0**.**112**	0.134	0.129	0.129	13.633	29.515
	4	1.00	1.00	1.00	1.00	1.00	1.00	0.137	0.156	**0**.**127**	**0**.**127**	12.445	26.858

Bold numbers indicate an outstanding performance among the methods

**Table 2:** Results: $$\sigma _{z}=0.5$$

No.TFs	Scenario	Feature selection of genes											
		TP				TN				Ave			
		Pro	NW.P	EL	LA	Pro	NW.P	EL	LA	Pro	NW.P	EL	LA
5	1	0.98	0.76	0.42	0.42	0.93	0.69	0.87	0.87	**0**.**96**	0.72	0.65	0.64
	2	0.98	0.73	0.41	0.39	0.93	0.65	0.83	0.88	**0**.**95**	0.69	0.62	0.63
	3	0.99	0.67	0.41	0.40	0.93	0.73	0.85	0.86	**0**.**96**	0.70	0.63	0.63
	4	0.98	0.74	0.44	0.41	0.94	0.71	0.83	0.87	**0**.**96**	0.73	0.64	0.64
10	1	0.98	0.73	0.36	0.36	0.93	0.77	0.93	0.93	**0**.**95**	0.75	0.65	0.65
	2	0.97	0.77	0.36	0.34	0.93	0.81	0.93	0.93	**0**.**95**	0.79	0.64	0.64
	3	0.99	0.75	0.37	0.35	0.93	0.78	0.92	0.94	**0**.**96**	0.76	0.65	0.65
	4	0.97	0.71	0.35	0.34	0.94	0.85	0.94	0.94	**0**.**96**	0.78	0.64	0.64
25	1	0.98	0.74	0.34	0.34	0.93	0.96	0.97	0.97	**0**.**96**	0.85	0.65	0.65
	2	0.97	0.66	0.31	0.30	0.94	0.97	0.96	0.97	**0**.**96**	0.81	0.64	0.63
	3	0.98	0.72	0.33	0.32	0.94	0.97	0.97	0.97	**0**.**96**	0.84	0.65	0.65
	4	0.97	0.70	0.32	0.32	0.94	0.97	0.97	0.97	**0**.**95**	0.83	0.64	0.64
50	1	0.98	0.76	0.31	0.31	0.94	0.97	0.98	0.97	**0**.**96**	0.87	0.64	0.64
	2	0.96	0.76	0.29	0.28	0.94	0.98	0.98	0.98	**0**.**95**	0.87	0.63	0.63
	3	0.99	0.75	0.32	0.32	0.94	0.97	0.98	0.98	**0**.**96**	0.86	0.65	0.65
	4	0.98	0.74	0.30	0.31	0.94	0.97	0.98	0.98	**0**.**96**	0.86	0.64	0.64
100	1	0.98	0.75	0.31	0.30	0.95	0.99	0.99	0.99	**0**.**96**	0.87	0.65	0.65
	2	0.96	0.74	0.28	0.27	0.95	0.98	0.99	0.99	**0**.**96**	0.86	0.63	0.63
	3	0.98	0.74	0.30	0.29	0.95	0.99	0.99	0.99	**0**.**96**	0.87	0.64	0.64
	4	0.98	0.78	0.28	0.28	0.95	0.98	0.99	0.99	**0**.**96**	0.88	0.63	0.63

No.TFs	Scenario	Feature selection of edges						Prediction accuracy					
		TP		TN		Ave		MSE					
		Pro	NW.P	Pro	NW.P	Pro	NW.P	Pro	NW.P	EL	LA	XGB	NN
5	1	1.00	1.00	1.00	0.92	1.00	0.96	**0**.**522**	0.558	0.536	0.531	9.596	2.991
	2	1.00	1.00	1.00	0.92	1.00	0.96	0.551	0.583	**0**.**550**	0.551	9.169	3.001
	3	1.00	1.00	1.00	0.92	1.00	0.96	**0**.**539**	0.588	0.550	0.546	11.543	3.633
	4	1.00	1.00	1.00	0.93	1.00	0.96	**0**.**538**	0.579	0.607	0.595	10.022	3.520
10	1	1.00	1.00	1.00	0.96	1.00	0.98	**0**.**552**	0.597	0.612	0.612	10.493	3.994
	2	1.00	1.00	1.00	0.96	1.00	0.98	0.541	0.566	**0**.**523**	0.524	8.880	3.960
	3	1.00	1.00	1.00	0.96	1.00	0.98	**0**.**551**	0.573	0.577	0.573	11.017	4.734
	4	1.00	1.00	1.00	0.96	1.00	0.98	**0**.**540**	0.571	0.605	0.602	9.706	4.816
25	1	1.00	1.00	1.00	0.98	1.00	0.99	0.597	0.621	0.567	**0**.**566**	11.130	6.067
	2	1.00	1.00	1.00	0.98	1.00	0.99	**0.552**	0.580	0.608	0.610	10.151	6.256
	3	1.00	1.00	1.00	0.98	1.00	0.99	**0.519**	0.538	0.613	0.612	12.434	7.857
	4	1.00	1.00	1.00	0.98	1.00	0.99	**0.552**	0.579	0.623	0.623	10.790	7.508
50	1	1.00	1.00	1.00	0.99	1.00	1.00	**0**.**600**	0.638	0.637	0.637	10.549	12.227
	2	1.00	1.00	1.00	0.99	1.00	1.00	**0**.**570**	0.598	0.639	0.626	9.595	11.736
	3	1.00	1.00	1.00	0.99	1.00	1.00	**0**.**579**	0.601	0.564	0.565	12.086	14.921
	4	1.00	1.00	1.00	0.99	1.00	1.00	**0**.**537**	0.559	0.612	0.617	10.722	14.226
100	1	1.00	1.00	1.00	1.00	1.00	1.00	0.630	0.647	0.612	**0**.**609**	12.256	23.636
	2	1.00	1.00	1.00	1.00	1.00	1.00	0.682	0.684	**0**.**631**	0.6338	11.634	21.663
	3	1.00	1.00	1.00	1.00	1.00	1.00	**0**.**590**	0.604	0.617	0.618	14.334	29.831
	4	1.00	1.00	1.00	1.00	1.00	1.00	**0**.**582**	0.585	0.645	0.639	12.218	27.657

Bold numbers indicate an outstanding performance among the methods

**Table 3:** Results: $$\sigma _{z}=1$$

No.TFs	Scenario	Feature selection of genes											
		TP				TN				Ave			
		Pro	NW.P	EL	LA	Pro	NW.P	EL	LA	Pro	NW.P	EL	LA
5	1	0.99	0.71	0.43	0.41	0.90	0.71	0.84	0.86	**0**.**95**	0.71	0.64	0.64
	2	0.98	0.79	0.43	0.39	0.92	0.61	0.86	0.90	**0**.**95**	0.70	0.64	0.65
	3	0.99	0.78	0.44	0.42	0.91	0.63	0.84	0.88	**0**.**95**	0.71	0.64	0.65
	4	0.97	0.70	0.42	0.41	0.90	0.65	0.87	0.87	**0**.**94**	0.68	0.64	0.64
10	1	0.98	0.69	0.40	0.37	0.90	0.79	0.91	0.94	**0**.**94**	0.74	0.65	0.65
	2	0.96	0.75	0.35	0.35	0.92	0.75	0.94	0.92	**0**.**94**	0.75	0.65	0.64
	3	0.98	0.71	0.39	0.38	0.92	0.78	0.93	0.93	**0**.**95**	0.74	0.66	0.66
	4	0.96	0.71	0.35	0.36	0.92	0.84	0.93	0.92	**0**.**94**	0.77	0.64	0.64
25	1	0.98	0.74	0.33	0.32	0.92	0.96	0.97	0.97	**0**.**95**	0.85	0.65	0.65
	2	0.97	0.73	0.33	0.32	0.92	0.97	0.96	0.97	**0**.**95**	0.85	0.64	0.64
	3	0.98	0.72	0.33	0.32	0.92	0.96	0.97	0.97	**0**.**95**	0.84	0.65	0.65
	4	0.97	0.65	0.32	0.31	0.92	0.97	0.96	0.96	**0**.**95**	0.81	0.64	0.64
50	1	0.98	0.71	0.32	0.31	0.93	0.98	0.98	0.98	**0**.**95**	0.84	0.65	0.64
	2	0.97	0.69	0.29	0.28	0.93	0.98	0.98	0.98	**0**.**95**	0.83	0.63	0.63
	3	0.98	0.76	0.31	0.30	0.93	0.98	0.98	0.98	**0**.**96**	0.87	0.64	0.64
	4	0.98	0.75	0.31	0.31	0.93	0.98	0.98	0.98	**0**.**95**	0.86	0.64	0.64
100	1	0.98	0.76	0.28	0.28	0.94	0.99	0.99	0.99	**0**.**96**	0.87	0.63	0.64
	2	0.96	0.75	0.26	0.26	0.94	0.99	0.99	0.99	**0**.**95**	0.87	0.62	0.63
	3	0.98	0.73	0.28	0.27	0.94	0.99	0.99	0.99	**0**.**96**	0.86	0.63	0.63
	4	0.97	0.74	0.28	0.28	0.94	0.99	0.98	0.98	**0**.**95**	0.86	0.63	0.63

No.TFs	Scenario	Feature selection of edges						Prediction accuracy					
		TP		TN		Ave		MSE					
		Pro	NW.P	Pro	NW.P	Pro	NW.P	Pro	NW.P	EL	LA	XGB	NN
5	1	1.00	1.00	1.00	0.92	1.00	0.96	**1**.**177**	1.197	1.128	1.129	10.422	3.691
	2	1.00	1.00	1.00	0.93	1.00	0.96	**1**.**053**	1.102	1.204	1.202	8.913	3.725
	3	1.00	1.00	1.00	0.92	1.00	0.96	**1**.**074**	1.121	1.308	1.300	11.849	4.357
	4	1.00	1.00	1.00	0.92	1.00	0.96	**1**.**052**	1.061	1.179	1.167	10.328	4.481
10	1	1.00	1.00	1.00	0.96	1.00	0.98	**1**.**071**	1.121	1.219	1.200	10.942	4.846
	2	1.00	1.00	1.00	0.96	1.00	0.98	1.121	1.183	**1**.**104**	1.109	9.856	4.901
	3	1.00	1.00	1.00	0.96	1.00	0.98	**1.150**	1.213	1.225	1.221	11.782	5.420
	4	1.00	1.00	1.00	0.96	1.00	0.98	**1**.**034**	1.089	1.254	1.236	10.697	5.556
25	1	1.00	1.00	1.00	0.98	1.00	0.99	**1**.**123**	1.195	1.188	1.194	11.915	6.768
	2	1.00	1.00	1.00	0.98	1.00	0.99	**1**.**021**	1.089	1.199	1.190	10.609	6.700
	3	1.00	1.00	1.00	0.98	1.00	0.99	**1**.**061**	1.113	1.266	1.264	12.294	8.391
	4	1.00	1.00	1.00	0.98	1.00	0.99	**1**.**112**	1.153	1.216	1.217	11.123	8.465
50	1	1.00	1.00	1.00	0.99	1.00	1.00	**1**.**080**	1.113	1.299	1.294	11.649	12.813
	2	1.00	1.00	1.00	0.99	1.00	1.00	**1**.**103**	1.132	1.218	1.221	10.737	12.295
	3	1.00	1.00	1.00	0.99	1.00	1.00	**1**.**089**	1.114	1.278	1.270	13.227	15.542
	4	1.00	1.00	1.00	0.99	1.00	1.00	**1**.**158**	1.190	1.266	1.252	11.878	14.920
100	1	1.00	1.00	1.00	1.00	1.00	1.00	1.210	**1.189**	1.191	1.192	13.263	24.151
	2	1.00	1.00	1.00	1.00	1.00	1.00	**1**.**169**	1.183	1.244	1.240	12.117	22.707
	3	1.00	1.00	1.00	1.00	1.00	1.00	1.199	**1**.**141**	1.233	1.232	15.166	30.391
	4	1.00	1.00	1.00	1.00	1.00	1.00	1.187	**1**.**158**	1.218	1.209	13.138	27.887

Bold numbers indicate an outstanding performance among the methods

For each scenario, we considered the number of TFs (*T*) as 5, 10, 25, 50, and 100 (No. TFs). We chose the optimal regularization parameter combination that minimized the following mean squared error computed from the validation dataset.

17 $$\begin{aligned} MSE=\frac{1}{n_{{\mathbb {V}}}}\sum _{i \in {\mathbb {V}}}(z_{i}-{\hat{z}}_{i})^{2} \end{aligned}$$

where ${\hat{z}}_{i}=\hat{\varvec{\theta }}^{T}\hat{{\varvec{B}}}^{T}{\varvec{x}}_{i}$, ${\mathbb {V}}$ and $n_{{\mathbb {V}}}$ are the set of indexes and sample size of validation dataset, respectively.

Our method was evaluated by comparing the prediction model based on an independently estimated network with prediction (NW.P), where the gene network was first estimated using the lasso. Then, the prediction of the response variable was based on the estimated network. We also considered prediction based on expression levels rather than networks, i.e. ${\varvec{X}}$ was used as an input of the prediction model. For these predictions, the lasso (LA), elastic net (EL), XGBoost (XGB) and neural network (NN) were used. For neural network, we used the fully-connected feed-forward neural network with a hidden layer based on ReLU activation function.

We compared the prediction results based on the prediction accuracy (mean squared error of the test sets) and the feature selection accuracy (true positive rates, true negative rates, and their average) of $\varvec{\theta }$ in the prediction model and of ${\varvec{B}}$ in the network estimation. Table 1 shows the feature selection accuracies of the genes in the prediction model and the network edges, and the prediction accuracy for $\sigma _{z}=0.1$. The column “Feature selection of genes” indicates the true positive rates, true negative rates, and their average for $\varvec{\theta }$. The feature selection accuracies for ${\varvec{B}}$ are given in column “Feature selection of edges”. The average mean square errors for 50 datasets are given as the prediction accuracy in the column “MSE”. We also show the results for $\sigma _{z}=0.5$ and $\sigma _{z}=1$ in Tables 2 and 3, respectively.

As shown in Table 1, the proposed method shows effective performance for feature selection in the prediction model (i.e. $\varvec{\theta }$). Although there were not large differences in the true negative rate, our method shows outstanding performance for the true positive rate. Other methods show poor results for true positive results. Our results demonstrate effective overall feature selection and gene selection in the prediction model (i.e., the average of the true positive and negative rates). The outstanding feature selection results in a prediction model (i.e. $\varvec{\theta }$), which can be also seen for $\sigma _{z}=0.5$ and $\sigma _{z}=1$, as shown in Tables 2 and 3. Furthermore, the PredictiveNetwork provides effective gene network estimation, i.e. effective edge selection results (i.e. ${\varvec{B}}$) were observed for all scenarios. Thus, our method provides efficient prediction accuracy overall. Although the predictive results are not very different between methods (i.e., Pro, NW.P, EL and LA), our strategy shows low prediction error in most scenarios.

We also illustrate the performances of the methods for various proportions of training dataset, i.e., 50%, 60%, 70% and 80% of *n*. We generate datasets from the scenarios for 30 TFs, $\sigma =1$ and $n=300$ and consider equal size of validation and test datasets. Table 4 shows the results of feature selection of genes (i.e., average of true positive and true negative for $\varvec{\theta }$) and prediction accuracy, where “Scn*X*” indicates the scenario *X*.

**Table 4:** Simulation studies for various proportion of training dataset

Methods	Proportion (%)	Ave of TP and TN for $\varvec{\theta }$				MSE			
		Scn1	Scn2	Scn3	Scn4	Scn1	Scn2	Scn3	Scn4
Pro	50	0.94	0.94	0.94	0.94	1.139	1.170	1.109	1.096
	60	0.95	0.95	0.95	0.95	1.152	1.036	1.085	1.113
	70	0.95	0.95	0.95	0.95	1.100	1.099	1.036	1.035
	80	0.94	0.95	0.94	0.95	1.047	1.110	0.962	1.124
NW.P	50	0.85	0.88	0.82	0.83	1.134	1.160	1.103	1.087
	60	0.87	0.82	0.84	0.83	1.149	1.068	1.107	1.120
	70	0.85	0.84	0.87	0.85	1.098	1.093	1.042	1.062
	80	0.83	0.87	0.89	0.85	1.032	1.106	0.966	1.145
EL	50	0.66	0.65	0.64	0.64	1.169	1.215	1.129	1.111
	60	0.65	0.63	0.64	0.64	1.200	1.107	1.142	1.160
	70	0.65	0.64	0.64	0.64	1.133	1.140	1.075	1.094
	80	0.65	0.64	0.65	0.64	1.086	1.142	0.983	1.173
LA	50	0.65	0.64	0.64	0.63	1.168	1.213	1.128	1.110
	60	0.65	0.63	0.64	0.63	1.193	1.105	1.137	1.155
	70	0.65	0.64	0.64	0.64	1.134	1.137	1.077	1.100
	80	0.65	0.64	0.65	0.64	1.073	1.139	0.990	1.163

As shown in Table 4, the feature selection results are not significantly affected by the proportion of training datasets. On the other hand, the prediction error (i.e., MSE) is getting larger as the proportion of training datasets gets smaller, in overall. The loss of prediction accuracy as the training datasets get smaller proportion can be seen in the results of not only our method but also existing approaches. Although the proposed method suffers from the loss of accuracy in small proportion of training set, superiority of the PredictiveNetwork can also be confirmed for various proportions of training datasets. In short, the proposed method provides effective results for gene network estimation, feature selection, and accuracy of prediction models constructed by incorporating network biology.

## Anti-gastric cancer drug sensitivity-predictive gene network analysis

To illustrate the proposed method, we applied our method to estimate drug sensitivity-predictive gene networks. We used a publicly available large scale pharmacogenomic data set, i.e. the “Sanger Genomics of Drug Sensitivity in Cancer (GDSC) dataset from the Cancer Genome Project”. The gene expression data (Cell_line_RMA_proc_basalExp.txt) and drug sensitivity data given as the half-maximal inhibitory concentration (IC50) and the Z-score for 345 compounds (GDSC1_fitted_dose_response_25Feb20.xlsx) are obtained from the GDSC dataset (https://www.cancerrxgene.org/downloads/bulk_download). We focused on FDA-approved drugs for stomach (gastric) cancer (https://www.cancer.gov/about-cancer/treatment/drugs/stomach). Among the 18 approved drugs, we considered four drugs: doxorubicin, Mitomycin-c, 5-Fluorouracil (5-FU), and Docetaxel that have drug sensitivity values in GDSC dataset. The expression levels of 10% of the genes (976 genes) with the highest variance in all cell lines were used for drug sensitivity-predictive network estimation. For each drug, we matched the expression levels and drug sensitivity (Z-score of IC50 value) for each cell line. The matching returned 948, 855, 891, and 948 cell lines consisting of around 30 cancer types for doxorubicin-, mitomycin-c-, 5-FU-, and docetaxel-sensitive predictive network estimation, respectively.

**Fig. 1:**
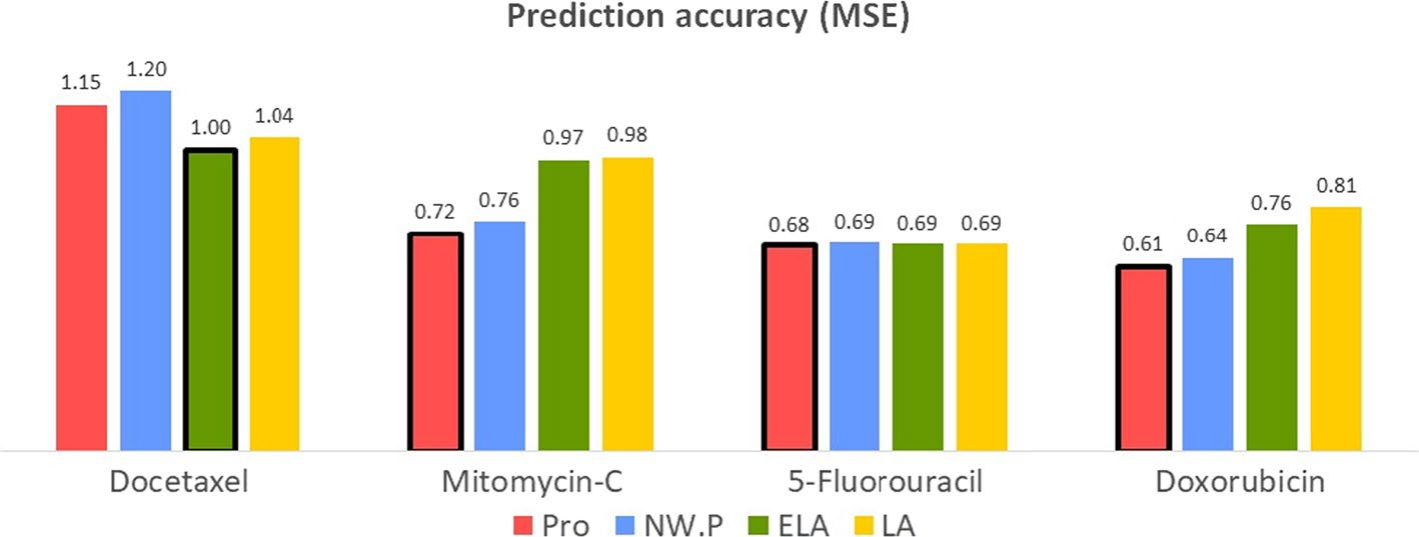
Prediction accuracy of anti-cancer drugs for gastric cancer

The prediction model consisted of 80%, 10%, and 10% of cell lines for the training, validation, and test datasets. Similar to the simulation study, we evaluated the prediction accuracy of our method by comparing the prediction results based on separately estimated networks (NW.P) and gene expression based prediction by lasso (LA) and elastic net (EL). The prediction results of the gastric cancer drug sensitivities are given in Fig. 1.

The proposed method shows effective results for predicting sensitivity to Mitomycin-c, 5-FU, and Docetaxel, while the elastic net showed outstanding performance for doxorubicin.

We next considered drug response predictive marker identification for gastric cancer. Genes having non-zero coefficient values ($\varvec{\theta }$) were considered as drug response predictive markers. Markers identified for all four drugs were considered as common markers that predict responses to anti-gastric cancer drugs. Table 5 shows the common markers, where the columns “ Gastric cancer drug” and “Gastric cancer” indicate evidence related to the mechanism of the anti-cancer drugs and gastric cancer, respectively.

**Table 5:** Evidences of the identified gastric cancer drug markers

Genes	Gastric cancer drug	Gastric cancer
*AKR1C1*	[18–24]	[19, 21, 25, 26]
*ZG16B1*	[27, 28]	[29]
*CRYAB*	[30]	[31–33]
*ZNF204P*	–	–
*C1QL1*	–	–
*TMEM139*	–	[34]
*PEG10*	[35–37]	[35, 38]

As shown in Table 5, more than half of the identified common markers were previously identified as markers for anti-cancer drugs and gastric cancer. *AKR1C1* is a well-known marker of drug resistance. The mechanism of *AKR1C1* underlying the acquired anti-cancer drug resistance has drawn a large amount of attention.

*Marker for anti-cancer drugs*

- *AKR1C1*
  Activation of the *Nrf2/AKR1C* axis contributes to oxaliplatin resistance in TSGH-S3 cells. Manipulating *Nrf2/AKR1Cs* activity may be useful for managing oxaliplatin-refractory gastric cancer [18]. The *AKR1C* family is involved in chemotherapy resistance in stomach, colon, lung, and brain cancers [19]. Furthermore, *AKR1C1* and *AKR1C3* inactivate doxorubicin cytotoxicity and are involved in oxaliplatin-resistant gastric cancer. *IL-6*, *AKR1C1*, and *AKR1C3* are the top 3 upregulated genes in TSGH-S3 human gastric carcinoma cells. A specific inhibitor of *AKR1C1* and *AKR1C3* was used to enhance cisplatin-induced cisplatin-resistance in signet ring cell gastric carcinoma (SRCGC) cells [19]. Genes from the *AKR1C* family are associated with resistance to CDDP and 5-FU. Controlling these genes enhances sensitivity to anti-cancer drugs by inhibiting cellular activity in drug-resistant cancer cells. Overexpression of *AKR1C* family was observed upon the acquisition of doxorubicin resistance. Various *AKR* family members are among the most differentially expressed genes upon the acquisition of doxorubicin resistance [20]. These findings suggest that *AKRs* may play a key role in doxorubicin resistance. Many anti-cancer drugs, e.g., doxorubicin, daunorubicin, and haloperidol, are metabolized by carbonyl-reducing enzymes including *AKR1A1, AKR1B1, AKR1B10, AKR1C1, AKR1C2*, and *AKR1C3*. The *aldo-keto reductase (AKR)* superfamily is also involved in the development of drug resistance in cancer cells [39]. *AKR1C1* and *AKR1C3* play a key role in cisplatin resistance in SRCGC by regulating redox-dependent autophagy. Further, *AKR1C1* is a crucial regulator of cisplatin-resistance in head and neck squamous cell carcinoma (HNSCC) and is a poor prognostic factor for HNSCC patient death [21]. Finally, *AKR1C1* is upregulated by *IL-6* and *Nrf2* and promotes acquired cisplatin-resistance in metastatic ovarian and gastric cancer cells. The overexpression of *AKR1B1, AKR1C1, AKR1C2*, and *AKR1C3* is responsible for the early appearance of doxorubicin drug resistance [22]. Sensitivity to cisplatin, cis-diamminedichloroplatinum (CDDP), and 5-FU is restored when *AKR1C1, AKR1C2, AKR1C3*, and *AKR1C* were knocked down. Inhibiting *AKR1C* family genes enhances sensitivity to CDDP and 5-FU [23]. *AKR1C1* plays an crucial role in drug resistance in bladder cancer cells [24]. Overexpression of *AKR* genes in cancer cells that are resistant to chemotherapeutic agents (i.e., cisplatin, doxorubicin, duanorubciin, mitomycin, emozolomide, cyclophosphosphamide, and oracin) is common [40]. Resistance to enzalutamide is caused by *AKR1C3* overexpression.
- *CRYAB*
  *CRYAB* protein levels are significantly reduced after doxorubicin treatment [30]. PDCryab1, a peptide from *CRYAB*, is a candidate for protecting the myocardium against doxorubicin-induced cell apoptosis.
- *PEG10*
  Knocking down *PEG10* enhances the sensitivity of MKN7 cells (human gastric adenocarcinoma cells) to docetaxel [35]. Inhibiting *PEG10* expression enhances the effect of 5-FU on apoptosis, and *PEG10* is upregulated in cases treated with neoadjuvant docetaxel. [36].

*Markers for gastric cancer*

- *AKR1C1*
  *AKR1C1* is a potential biomarker and therapeutic target for gastric cancer [25].
- *CRYAB*
  *CRYAB* is a therapeutic target for gastric cancer [31]. *CRYAB* is a prognostic biomarker and therapeutic target in human solid tumors. The role of *CRYAB* in anti-cancer invasion and metastasis via epithelial-mesenchymal transition (EMT) was recently uncovered [32]. Increased *CRYAB* expression is associated with poor overall survival in digestive system cancer patients. *CRYAB* contributes to gastric cancer cell migration and invasion via EMT, which is mediated by the *NF*-$\kappa$*B* signaling pathway. Tao et al. [33] demonstrated that high *CRYAB* levels are related to angiogenesis and poor prognosis in gastric cancer.
- *TMEM139*
  *TMEM139* was identified as a differentially expressed gene in intestinal metaplasia that does not progress to gastric cancer [34].
- *PEG10*
  *PEG10* is a high lymph node ratio-associated gene whose expression is positively correlated with pathological stage III gastric cancer [35]. Knockdown of *PEG10* suppresses proliferation, invasion, and decreases chemo-resistance in gastric cancer cells. Silencing lncRNA *PEG10* inhibits the occurrence and progression of gastric cancer [38].
- *ZG16B1*
  *ZG16B* is a tumorigenic factor and diagnostic marker in pancreatic, gastric, colon, ovarian, oral squamous cell, and cervical carcinoma [29].

**Fig. 2:**
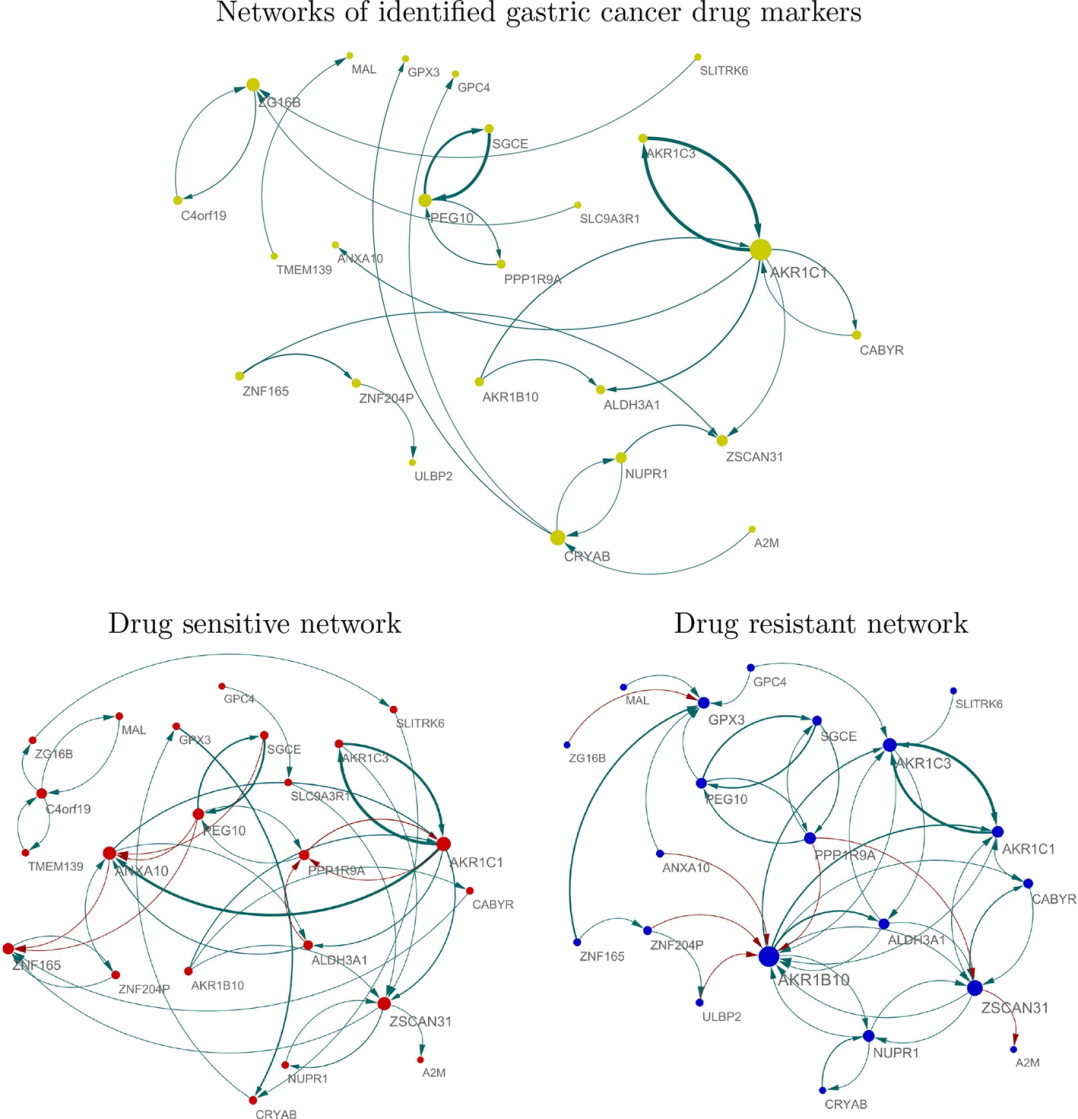
Gene networks for the identified gastric cancer drug markers in drug-sensitive and -resistant cell lines . Edge thickness represents the strength of effect of regulator on target genes (i.e., $|\beta |$) and color indicates sign of the effect (red: “-” and blue: “+”). Node size represents degree of connectivity (i.e., hubness) of each gene in the networks

To uncover common regulatory systems involved in gastric drug responses, we constructed a gene network based on the target and regulator genes of the common markers. We consider the regulator (target) genes of the common markers in the estimated networks for more than one drug as common regulators (target) of the identified markers. Hereafter, we refer the common genes, common regulator and target genes as identified gastric cancer drug markers. For the identified markers, we extract their networks from $\hat{{\varvec{B}}}^{\text {doxorubicin}}$, $\hat{{\varvec{B}}}^{\text {mitomycin-c}}$, $\hat{{\varvec{B}}}^{\text {5-FU}}$, $\hat{{\varvec{B}}}^{\text {docetaxel}}$ estimated for the doxorubicin, mitomycin-c, 5-Fluorouracil (5-FU), and docetaxel -networks, respectively. To clearly visualize the network, we consider edges having absolute values of $B_{jl}$ greater than 0.1. We then compute median of the edge sizes (${\hat{B}}_{jl}^{\text {doxorubicin}}$, ${\hat{B}}_{jl}^{\text {mitomycin-c}}$, ${\hat{B}}_{jl}^{\text {5-FU}}$, ${\hat{B}}_{jl}^{\text {docetaxel}}$ that indicate strength of effect of gene *l* on gene *j*, i.e., $\varvec{x_{l}} \rightarrow {\varvec{x}}_{j}$), and we let the median of edge size as $B^{CM}_{jl}$. Top of Fig. 2 shows the gene regulator network described by $B^{CM}_{jl}$ .

*AKR1C1*, a crucial marker of drug resistance, is a hub gene in gastric cancer drug sensitivity-predictive gene networks. Strong interaction between *AKR1C1* and *AKR1C3* was present in the network, which implies that *AKR* family genes are tumorigenic factors and diagnostic markers for gastric cancer. Further, our results suggest that these genes may be activated by molecular interactions rather than the activity of a single gene. There is abundant evidence that the *AKR1C* family, including *AKR1C1* and *AKR1C3*, plays a key role in drug resistance of gastric cancer. These findings imply that our method provides biologically reliable results and that the constructed network for gastric cancer drugs may have crucial information to uncover mechanism-related therapeutic resistance and chemotherapy effectiveness. Importantly, these factors cannot be identified without considering molecular interactions.

To identify gastric cancer drug-sensitive and -resistant molecular interactions, we estimated drug-sensitive and -resistant networks based on identified gastric cancer drug markers. We extracted data from 400 drug-sensitive and -resistant cell lines. This data corresponded to the 400 largest (resistant) and 400 smallest (sensitive) drug sensitivity values of each drug. We then extracted the overlapping drug-sensitive and -resistant) cell lines for the four drugs (95 drug sensitive and 95 drug resistant cell lines were extracted) and estimated gene networks based on these cell lines. For each 95 drug sensitive and 95 drug resistant cell lines, we estimate drug sensitive and resistant gene networks (i.e., $NW^{st}$ and $NW^{rs}$) consisting of the 10% of the genes (976 genes) having the highest variance by using the lasso, i.e., the regulatory system between target gene ${\varvec{y}}_{j}$ and regulator genes ${\varvec{x}}_{l}$ is estimated as $\beta _{jl}$ by (2) with $\lambda _{1}=0$. The gene networks are described by $\hat{{\varvec{B}}}^{st}$ and $\hat{{\varvec{B}}}^{rs}$ for drug sensitive and resistant cell lines, respectively. From $\hat{{\varvec{B}}}^{st}$ and $\hat{{\varvec{B}}}^{rs}$, we extract the network consisting of the identified gastric cancer drug markers, where the edges having absolute values of $B_{jl}$ greater than 0.1 are only extracted to clearly visualize the networks The bottom of Fig. 2 shows the gene networks of the identified gastric cancer drug markers for drug-sensitive and -resistant cell lines.

Our results show that the identified gastric cancer drug markers have different regulatory systems in drug-sensitive and -resistant cell lines. *AKR1C1* and *AKR1C3* show strong interactions in both drug-sensitive and -resistant cell lines. The hubness of *AKR1B10* is a characteristic of drug-resistant gastric cancer cells. Indeed, the hubness of *AKR1B10* was significantly smaller in drug-resistant cells compared to sensitive cell lines. The hubness of *AKR1C3* also became smaller in drug-sensitive cell lines compared with networks estimated from drug-resistant cell lines. The activity of *AKR* family genes in drug resistant cell lines was strongly supported by previous studies [19–24, 39, 40].

**Fig. 3:**
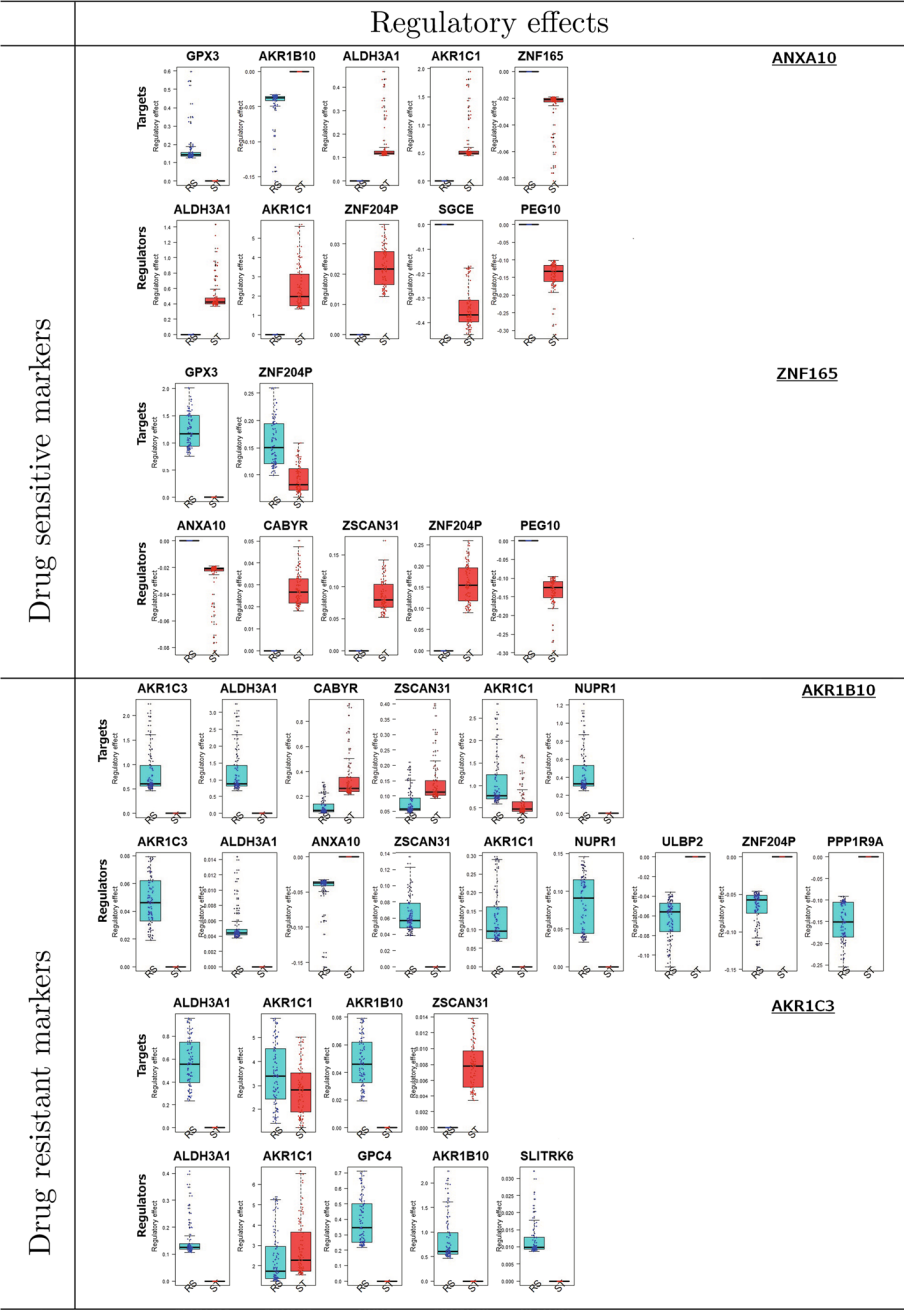
Regulatory effects of the gastric cancer drug -sensitivity and -resistance markers. The regulatory effects of genes indicate ${\varvec{x}}^{st}_{l}{\hat{\beta }}^{st}_{jl}$ and ${\varvec{x}}^{rs}_{l}{\hat{\beta }}^{rs}_{jl}$ for drug sensitive and resistant cell lines, respectively, where ${\hat{\beta }}^{st}_{jl}$ and ${\hat{\beta }}^{rs}_{jl}$ are estimated in drug sensitive and resistant networks in Fig. 2. “Targets” indicates regulatory effect of the identified markers on their target genes and “Regulators” indicates regulatory effect of the genes on the identified markers in drug sensitive and resistant networks (i.e., $NW^{st}$ and $NW^{rs}$)

In contrast, the hubnesses of *ANXA10* and *ZNF165* are characteristics of drug-sensitive gastric cancer cells. *ANXA10* and *AKR1C1* showed strong interactions in drug-sensitive cell lines, while their interaction disappeared in drug-resistant cell lines. The hubness of *ZNF165* becomes weaker from the drug sensitive to resistant cell lines. Thus, high activities of *ANXA10* and *ZNF165* can be considered as signatures of drug-sensitive cell lines. It has been identified that *ANXA10* is a crucial marker of gastric cancer and its related mechanism for drug sensitivity has been uncovered as follows. *ANXA10*, a novel gastric marker, shows extensive tissue and subclonal heterogeneity of dual stomach-intestinal cell states [41]. Additionally, *ANXA10* is significantly upregulated in gastric carcinoma and downregulated in gastric carcinogenesis [42]. Overexpression of *ANXA10* in MKN-1 human gastric adenosquamous carcinoma cells leads to cell growth and increased apoptotic cells. These results suggest that *ANXA10* plays a crucial role as a tumor suppressor in gastric cancer cells by restraining cell growth and inducing basal apoptosis. In around half of gastric cancer cases, *ANXA10* is detected. Loss of *ANXA10* is significantly correlated with disease progression and poor clinical outcomes in gastric cancer [43]. Repressed cell growth was observed in *ANXA10*-knockdown human gastric organoids. Hierarchical clustering showed that *KLK6* and *ANXA10* are enriched in cancer organoids that showed higher sensitivity to erlotinib [44]. Overexpression of *ANXA10* in human epithelial cancer cells increases sensitivity to doxorubicin-induced apoptosis and reduces clonogenic ability [45]. *ZNF165* is a novel cancer antigen capable of eliciting humoral immune responses and is involved in tumour biology [46]. Further, *ZNF165* is expressed in gastric cancer, colon cancer, and non-small-cell lung carcinoma. Previous studies strongly support our data-driven results that high *ANXA10* activity is a characteristic of drug-sensitive gastric cancer cells. Drug-sensitive and/or -resistance-specific molecular interactions may be crucial clues for uncovering drug resistance/senstivity mechanisms. We show regulatory effects for drug-sensitive and -resistance-specific markers, where the regulatory effect is computed by the combined regulator expression level and the effect of the regulator on its target gene. For the drug sensitive and resistant cell lines, the regulatory effect of $l^{th}$ regulator gene on $j^{th}$ target gene is described by ${\varvec{x}}^{st}_{l}{\hat{\beta }}^{st}_{jl}$ and ${\varvec{x}}^{rs}_{l}{\hat{\beta }}^{rs}_{jl}$, respectively, where ${\varvec{x}}^{st}_{l}$ (${\varvec{x}}^{rs}_{l}$) is the expression levels of gene *l* in drug sensitive (resistant) cell lines obtained from GDSC dataset and ${\hat{\beta }}^{st}_{jl}$ (${\hat{\beta }}^{rs}_{jl}$) is the estimated effect of gene *l* on gene *j* in drug sensitive (resistant) network (i.e., $\hat{{\varvec{B}}}^{st}$ and $\hat{{\varvec{B}}}^{rs}$ which are estimated for Fig. 2). Thus, gene activity can be described by the regulatory effect. Figure 3 shows the regulatory effect of the identified gastric cancer drug-sensitive and -resistant markers on their targets (row: Targets) and the regulatory effect of their regulators on the identified markers (row: Regulators) in drug-sensitive and -resistant cell lines. As shown in Fig. 3, the identified drug sensitive markers *ANXA10* and *ZNF165* showed high activity in drug-sensitive cell lines, especially the effects of their regulators on *ANXA10* and *ZNF165* are disappeared in drug resistant cell lines (i.e., ${\hat{\beta }}^{rs}=0$). Especially, their regulators show a large regulatory effect on *ANXA10* and *ZNF165* in drug-sensitive cell lines. These effects become smaller in the drug-resistant cells. The drug resistance markers also showed clearly different activities between drug-sensitive and -resistant cell lines. The genes regulating *AKR1B10* act only in drug resistant cell-lines, and interactions between regulators and *AKR1B10* disappeared in drug-sensitive cells.

**Fig. 4:**
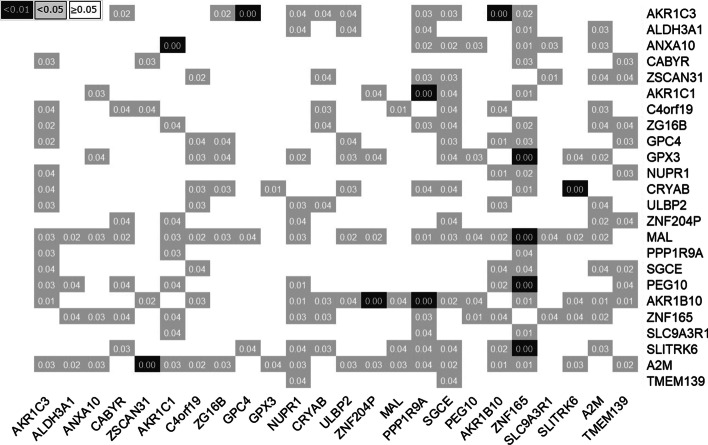
Significance of difference of molecular interactions between drug-sensitive and -resistant cell lines. The color indicates the significance of the interaction: white, grey, and black means *p* value$\ge 0.05$, *p* value$<0.05$ and *p* value$<0.01$, respectively

We then identify molecular interactions that show significantly different regulatory system between drug-sensitive and -resistant cell lines. We randomly selected 95 cell lines and estimated networks (permuted drug-sensitive networks) similar to the networks in the bottom of Fig. 2. We also estimated permuted drug-resistant networks based on 95 randomly-selected cell lines. The differences between the two networks were computed for 1000 randomly-selected permutation samples ($DF(pm), \quad pm=1,...,1000$). We computed the following permutation *p* value based on the difference between the two networks given in Fig. 2 ($DF_{true}$) and *DF*(*pm*),

18 $$\begin{aligned} p~\text {value}_{pm}=\frac{\sum _{pm=1}^{1000}{\varvec{I}}(|DF_{true}|<|DF(pm)|))}{1000} \end{aligned}$$

where ${\varvec{I}}(\cdot )$ is the indicator function. The $p~\text {value}_{pm}$ indicates the proportion that the absolute difference of edges in drug sensitive and resistant cell lines ($DF_{true}$) is smaller than the absolute differences computed from the 1000 permuted samples ($DF_{pm}$). The $p~\text {value}_{pm}$ indicates that two genes show extremely different regulatory system between drug sensitive and resistant cell lines.

Figure 4 shows the $p~\text {value}_{perm}$ of permutation test to test difference of the regulatory system of the identified gastric cancer drug markers, where the markers are considered as not only regulator (rows) but also target (columns) genes. The identified drug resistance marker *AKR1B10* showed significantly different regulatory effects on *AKR1C3*. Furthermore, another drug resistance marker, *ZNF165*, had several significantly different molecular interactions for its target genes (i.e., *GPX3*, *MAL, PEG10*, and *SLITRK6*). Further, the drug sensitivity markers *AKR1C3* and *ANXA10* had significantly different regulatory systems with their regulators (*AKR1C3*: *GPC4* and *PEG10*, *ANXA10*: *AKR1C1)*. In addition to *ANXA10, ZNF165, AKR1C1* and *AKR1B10*, we identified several more genes as common predictive markers for the four gastric cancer drugs (i.e., genes in the networks) that showed significant differences in the networks of drug sensitive and resistant cell lines (*p* value < 0.05). These results imply that identified gastric cancer drug markers have significantly different regulatory systems in the gene networks between the drug-sensitive and -resistant cell lines.

From these results, we suggest that the high activity of *AKR* family genes may be involved in acquired drug resistance. Thus, controlling suppressors of *AKR* family genes may enhance the sensitivity to gastric cancer drugs (Additional file 1: Table S1). Our results also suggest that loss of *ANXA10* activity may lead to drug resistance. Although little evidence for the role of *ZNF165* were found for mechanisms related to gastric cancer or anti-cancer drugs, it can be suggested that ZNF165 is a novel marker of gastric cancer drug responses. The controlling inducers of *ANXA10* and *ZNF165* may lead to enhanced drug sensitivity (Additional file 1: Table S1).

## Discussion

We introduced a novel computational strategy for response variable predictive gene network estimation. To identify biological mechanism-specific gene networks, we propose a model that consists of gene network estimation and prediction of a specific biological process. Furthermore, we incorporated network biology into the prediction model, which enabled the PredictiveNetwork to simultaneously perform gene network estimation and prediction. Our method estimates gene networks that achieves minimized prediction and network estimation errors. Thus, we can identify response prediction-specific characteristics of gene networks. Additionally, our method can construct prediction models based on crucial subnetworks involved in specific biological processes. These lead to effective interpretation of prediction results and biologically-reliable predictive marker identification.

Graph Attention Networks (GAN) [47] is a strategy that also performs network estimation and prediction, simultaneously. GAN is a neural network approach that leverages masked self-attentional layers based on similarity of node in neighborhoods. Thus, gene regulatory system can be described by clinical characteristic-specific self-attention network. On the other hand, the gene network estimation procedure of the PredictiveNetwork can be considered as clinical characteristic specific graphical gaussian modeling and the estimated gene regulatory network is given as weighted-directed adjacency matrix.

To illustrate the proposed strategy, we performed Monte Carlo simulations. The simulation results showed that the proposed strategy has outstanding performance for feature selection in gene network estimation and prediction. Our results also demonstrate excellent prediction accuracy. We applied the proposed PredictiveNetwork to estimate gene networks that are responsive to gastric cancer drugs. Using the GDSC dataset, we estimated doxorubicin, Mitomycin-C, 5-FU, and Docetaxel-responsive gene networks. The identified gastric drug response markers showed significantly different regulatory systems between drug-sensitive and -resistant cell lines. Combined with previous studies, the identified gastric drug response markers and drug sensitivity/resistance-specific markers have strong evidence for mechanisms related to anti-cancer drugs and gastric cancer. In particular, our results indicate that *AKR* family genes are likely drug resistance markers. We identified the drug sensitivity-specific activity of *ANXA10* and *ZNF162*, which is strongly supported by previous studies. Collectively, our results of GDSC data analysis suggest that the molecular interplay between *ARK* family genes and *ANXA10/ZNF162* activity play key role in the mechanisms underlying acquired resistance/sensitivity to gastric cancer drugs. Manipulating suppressors and induces of ARK family genes, *ANXA10*, and *ZNF162* may be a way to reduce drug resistance of cancer cell lines.

## Supplementary Information

**Additional file 1**. The list of suppressors and inducers for AKR1B10, AKR1C3, ANXA10 and ZNF165.

## Data Availability

The datasets are available from Sanger Genomics of Drug Sensitivity in Cancer (GDSC) dataset(https://www.cancerrxgene.org/). R script for PredictiveNetwork is available at https://drive.google.com/file/d/1DdxnWtHeYcF_6H4yQQYN3NTNGTRYtD_f/view?usp=sharing.
